# Process, Physicochemical Characterization and *In-Vitro* Assessment of Albendazole Microcrystals

**DOI:** 10.15171/apb.2017.050

**Published:** 2017-09-25

**Authors:** Vandana KR, Prasanna Raju Yalavarthi, Harini Chowdary Vadlamudi, Jagadesh Kumar Yadav Kalluri, Arun Rasheed

**Affiliations:** ^1^Pharmaceutics Division, Sree Vidyanikethan College of Pharmacy, A.Rangampet, Tirupati, IN-517102.; ^2^Pharmaceutics Division, Sri Padmavathi School of Pharmacy, Tiruchanur, Tirupati, IN-517503.; ^3^Pharmaceutics Division, PES College of Pharmacy, Bangalore, IN-560050.; ^4^Department of Phytopharmaceutics, Al-Shifa College of Pharmacy, Poonthavanam, IN-679325.

**Keywords:** Particle engineering, Stabilizers, Adsorption, Enthalpy, Dissolution efficiency, Surface energy

## Abstract

***Purpose:*** Albendazole is a poorly soluble drug which limits its oral bioavailability. The study was focussed to enhance the solubility by in-situ micronization.

***Methods:*** Albendazole microcrystals were prepared by solvent change method using gum karaya and hupu gum as stabilizing agents and the effect of each stabilizer on the prepared microcrystals were studied. FT-IR, DSC, XRD and SEM analysis were performed as a part of characterization studies. The formulations were evaluated for micromeritics, solubility and drug release. The microcrystals that had shown optimized properties were filled into suitable capsules.

***Results:*** The formulations showed reduction in particle size with uniform size distribution and three folds increase in drug release. The microcrystals had shown more than 100-folds increase in solubility compared to pure drug. Surface energy, enthalpy and crystalline nature of microcrystals were found to be reduced. Microcrystals containing gum karaya had shown more drug release. The filled-in capsules also showed increase in drug release rate. The solubility enhancement of albendazole microcrystals was mainly due to the surface adsorption of the stabilizing agents that led to reduction in surface energy and crystalline nature as substantiated by the DSC and XRD studies. The type of stabilizing agent had significant effect on dissolution rate. High affinity of albendazole with gum karaya led to faster drug release profiles.

***Conclusion:*** The study proved that in-situ micronization is an effective technique to enhance the solubility and dissolution rate of poorly soluble drugs like albendazole.

## Introduction

It is well known that solubility and dissolution rate are major factors that affect bioavailability of orally administered drugs. Dissolution rate plays a key role in attaining the suitable blood levels of drug candidates of BCS class II and IV. There are many techniques to enhance dissolution rate of poorly soluble drugs such as jet milling, solid dispersions, and liquisolid formulations which depends on increasing the specific surface area of particles to enhance dissolution rate. Micronization, a size reduction technique, is one of the most prominent and reliable methods to enhance drug solubility and dissolution rate. In this method, the particle size distribution is kept less than 10 μm, which increases surface area-to-volume ratio, dissolution rate and adherence to surface resulting in high dissolution in GI fluids. Micronization by milling is inefficient due to high energy input that lead to disruption of the crystal lattice causing enhanced electrostatic effects, broad particle size distribution and thermodynamic instability.^1-3^ In order to overcome above problems, various particle engineering techniques such as spray drying, super critical fluid (SCF) technologies and *in-situ* micronization, which facilitate production of drug in required particle size, has gained importance.

Spray drying and SCF are found to be less reliable owing to their prolonged processing conditions and expensive equipments, whereas, *in-situ* micronization, a novel approach was proved to be successful in enhancement of dissolution of celecoxib, gliclazide, betamethazone, prednisolone, budesonide, itraconazole and ketoconazole.^4-7^ In this technique, drug particles are prepared in micronized state during particle formation, without using any external processing conditions like mechanical force, temperature and pressure.^3,8,9^ Also, microcrystallization occurs with simultaneous surface modification with the help of stabilizing agents that hinders the newly formed surfaces and reduces the electrostatic forces formed during nucleation.

Albendazole (ABZ) is a widely used antiparasitic agent. It is practically insoluble in water with oral absorption about 1 - < 5%.^9^ Many attempts were made to enhance the bioavailability of albendazole with little or no success. When hydrophobic drugs are micronized, energy of newly formed particles increases due to formation of more number of hydrophobic surfaces. A stabilizing agent is therefore required to form hydrophilic protective layer around the newly formed surfaces spontaneously thereby preventing agglomeration by steric hinderance. Addition of stabilizing agents therefore enhances the effective surface area, wetting properties and stability of microcrystals.^9-11^

The present study was focussed on the *in-situ* micronization process by solvent change method to produce microcrystals of a model drug candidate, albendazole that belongs to BCS class II. In recent past, gum karaya (GK) and hupu gum (HG), naturally hydrophilic polysaccharides have shown their promise as carriers in modified drug delivery systems,^12-14^ stabilizing and film forming agents.^15-17^ Further it was also attempted to explore the suitability and applicability of GK and HG in optimizing solubility and dissolution rate of BCS class-II and IV drug candidates in association of *in-situ* micronization process.

## Materials and Methods

### Materials

Albendazole was gratis of M/s. A-Z Pharmaceuticals, Chennai, India and Hupu gum (grade I) was obtained from Girijan Co-operative Corporation, Visakhapatnam, India. Gum karaya (grade I) was purchased from S.D. Fine Chem. Ltd, Mumbai, India. Other reagents and solvents were of analytical and pharmaceutical grade.

### Methods

#### In-situ micronization

Albendazole microcrystals were prepared by solvent change method which follows Ostwald-Miers rule (Rasenack et al., 2004a). Thus, supersaturated solution of ABZ was prepared by dissolving excess amount of albendazole in 20 ml of anhydrous formic acid and filtered. The filtrate was used to prepare microcrystals. Excess amount (» 2.5%) of gum karaya (GK) and hupu gum (HG) were taken and equilibrated in distilled water overnight. The resultant solution forms the non-solvent solution for the preparation of albendazole microcrystals (ABZ-GK and ABZ-HG). Rapid mixing of solvent and non-solvent solutions in batch wise under continuous stirring resulted in spontaneous formation of micro-fine dispersion.^4,18,19^ The micro-fine dispersion was then filtered and dried for further evaluations. ABZ crystals without the addition of stabilizing agents (Untreated ABZ) were also prepared by solvent change method to compare solubility.

#### Saturation solubility studies

Saturated solutions were prepared by adding excess amount of pure ABZ, untreated ABZ and the prepared microcrystals separately into conical flasks, each containing 25 ml of distilled water and kept in shaker (Remi) for 72 h at room temperature.^20^ The content of each conical flask was then filtered through 0.45 mm nylon filter. The filtrate was then diluted with distilled water and assayed spectrophotometrically using UV-Visible Spectophotometer (UV-1700, Shimadzu) at 298 nm.

#### Microscopic images

A thin layer of albendazole and the microcrystals were spread in a cavity slide and covered with a cover slip. The slide was observed under microscope (Olympus, BX 51- P) with and without polarized light. Photomicrographs were taken at suitable magnifications.

#### Particle size analysis

The samples of ABZ and microcrystals were placed on a glass slide and the size of 500 particles was measured using a calibrated eyepiece micrometer. The size distribution curve was plotted and the mean particle diameter was calculated.

#### Determination of flow properties

The flow properties were predicted from the values of compressibility index, Hausner’s ratio and angle of repose.

#### Fourier Transform Infra-red Spectroscopy (FT-IR) 

The spectra of pure drug (ABZ) and the microcrystals were recorded in FT-IR spectrophotometer (Thermo-IR 200) using KBr pellet method under identical conditions. Each spectrum was derived from 16 single average scans obtained in the scanning region of 4000 - 400 cm^-1^ at 2 cm^-1^ resolution of scans.

#### Thermal analysis

Differential Scanning Calorimetry (DSC) thermograms of pure albendazole and the microcrystals were recorded using Shimadzu DSC-50. The samples were heated (0–300°C) at a heating rate of 10°C/min. The analysis was performed under nitrogen purge (20 ml/min). The samples were weighted into standard aluminum pans and an empty pan was used as reference. The obtained DSC graphs were interpreted from melting point and enthalpy.

#### X-Ray Diffraction (XRD) studies

XRD spectra of pure albendazole and microcrystals of ABZ-GK and ABZ-HG were recorded on SIEFERT 303, Germany, X-ray diffractometer using CuK_a_ radiation generated at 40 kV and 30 mA. The data were recorded over 2q range of 10 – 80° at a preset time of 0.2 seconds with 4°/min scanning speed. The relative intensity I/I_0_ corresponding to the 2q value were reported.

#### Drug content determination

The percent drug content was determined by dissolving known quantity of ABZ-GK and ABZ-HG microcrystals in anhydrous formic acid. The solution was filtered and assayed upon Beer dilutions. The experiment was done for three independent samples.

#### In-vitro dissolution studies

Dissolution study was conducted for pure drug, ABZ and prepared microcrystals using USP type-II apparatus with 0.1 N HCl as dissolution medium under identical conditions. Samples of 5 ml were withdrawn at specified time intervals over 120 min and filtered through 0.45 mm filter. The experiment was set for sink condition. The samples were assayed at 298 nm. The results were calculated as a mean of six independent observations. Dissolution parameters such as DE_60_, DE_90_, T_50_ and T_90_ were calculated to characterize the ABZ dissolution profiles. T_50_ and T_90_ points were measured at the time points of 50% and 90% of the drug dissolved. Wherein dissolution efficiency (DE) was calculated using following formula:^21^

(1) $$Dissolution\text{ Efficency (DE) }=\;\left[\frac{\int_{\text{0}}^{t}ydt}{y_{100}t}\right]x100$$

where y = amount of drug released at time (t)

#### Scanning Electron Microscopy (SEM)

Electron micrographs were derived using a scanning electron microscope (Jeol, JSM-840 A, Japan) operating at accelerating voltage of 25 kV. The test samples were mounted on alumina stubs using double-sided adhesive tape, coated with gold in HUS-5GB vacuum evaporator.

#### Preparation of albendazole capsules 

Microcrystals equivalent to 200 mg albendazole dose was encapsulated into size ‘1’capsules. Albendazole microcrystals filled in capsules (AMC) were compared with commercial albendazole capsules (CAC) (Gekare®) as well as pure drug filled in capsules (PAC).

#### Evaluation of capsules 

The filled-in capsules were subjected to weight variation and disintegration test as per USP specifications. The capsules AMC, CAC and PAC were subjected to *in-vitro* dissolution study. The dissolution parameters viz., DE_60_, DE_90_, T_50_ and T_90_ were calculated. To appreciate the possible release mechanism of ABZ from capsule units, the drug release data were fitted to various kinetic models.

## Results and Discussion

### Solubility studies

ABZ-GK and ABZ-HG microcrystals had shown 196 and 176-folds increase in solubility respectively compared to pure drug. Whereas, crystallization of albendazole without stabilizing agent (untreated ABZ) showed merely 2.4-folds increase in solubility. The results are presented in Table 1. *In-situ* micronization of ABZ was carried out by solvent change method. Gum karaya and hupu gum are hydrophilic natural polysaccharides which served as stabilizing agents in the present study. It is apparent from the results that the adsorption of stabilizers over newly formed crystal surfaces prevented crystal growth that eventually led to increase in effective surface area of wetting. Solubility of albendazole crystallized without stabilizing agent (untreated ABZ) was less due to absence of hydrophilic shield that eventually led to high surface energy and aggregation of newly formed crystals. The ABZ-GK microcrystals possessed highest solubility due to albendazole affinity with gum karaya that caused increased hydrophilic shielding compared to that of hupu gum.

**Table 1:** Evaluation of microcrystals (ABZ: Albendazole, GK:Gum karaya, HG:Hupu gum)

**Formulation**	^a^ **Solubility (mg/ml)**	**Particle size (µm)**	**Compressibility index (%)**	**Angle of repose (θ)**
ABZ	0.005±0.95	115.9±1.21	24.8±1.21	45.41±1.87
Untreated ABZ	0.012±1.02	-	-	-
ABZ-GK	0.978±0.45	44.9±0.12	13.7±1.46	30.07±1.01
ABZ-HG	0.882±1.24	44.7±0.26	15.3±0.09	32.4±1.07

Values are expressed as mean ± S.D. (n = 3): ^a^mean ± % R.S.D. (n = 3)

### Micromeritics

**Figure 1:**
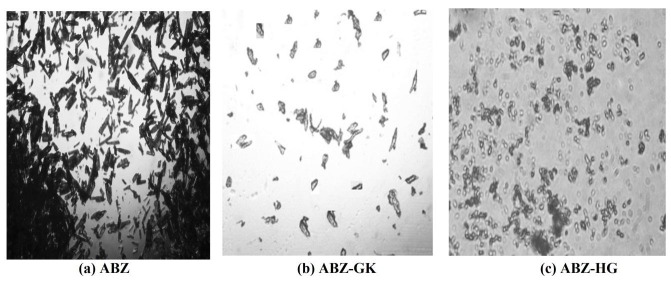
Microscopic images of (a) ABZ (b) ABZ-GK (c) ABZ-HG (ABZ: Albendazole, GK:Gum karaya, HG:Hupu gum)

Microcrystals prepared by *in-situ* micronization technique were evaluated for particle size, size distribution, compressibility index and angle of repose. The results are summarized in Table 1. Significant reduction in particle size was observed from the microscopic images as shown in Figure 1(a,b,c). Pure drug and ABZ-HG microcrystals had shown rod/rectangular shape and spherical shape respectively, whereas, ABZ-GK microcrystals are irregular and not shown any particular shape. Microcrystals showed uniform distribution with mean size of 40 µm. Compressibility index was observed in the range of 13-16% for microcrystals and the angle of repose values were between 30 and 33°. The obtained microcrystals were evaluated for micromeritic behavior. Adsorption of polysaccharide stabilizers onto the albendazole mirocrystal surfaces facilitated the uniform size distribution without undergoing electrostatic agglomeration. It was observed that the type of stabilizing agent had no significant effect on particle size. Lower values of compressibility index and angle of repose confirms that adsorption of stabilizing agents led to decrease in cohesiveness thereby improving flow properties of microcrystals compared to that of untreated albendazole.^22^

### FT-IR studies

The FT-IR spectra (Figure not shown) of pure albendazole and microcrystals showed that all samples were identical retaining the finger print region of albendazole. The principal absorption peaks of benzimidazole group around 3321.74 (NH stretching) and 2662.05 (C-N stretching) were retained in the microcrystals. Propyl-thio group which showed absorption peak around 1268 cm^-1^(S-CH_2_R deformation) was also not shifted. The absorption peak around 2953 cm^-1^ was observed in microcrystals without any deviation. Characterization studies were performed to analyze the interactions, if any, between the drug and the excipients. FT-IR studies had shown that all the samples were identical inferring no possible interactions at molecular level.

### Thermal analysis

**Figure 2:**
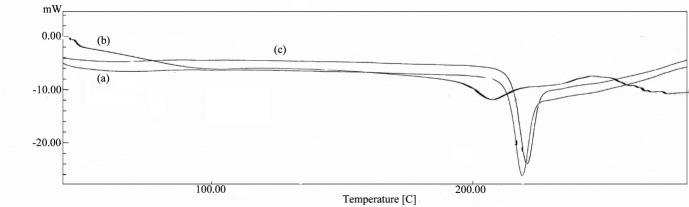
Comparative DSC thermograms of albendazole and microcrystals, (a) ABZ (b) ABZ-GK and (C) ABZ-HG (ABZ: Albendazole, GK:Gum karaya, HG:Hupu gum)

Thermograms of ABZ, ABZ-GK and ABZ-HG had shown various endothermic peaks with similar melting points shown in Figure 2. Reduction in the enthalpy was observed for the microcrystals compared to that of pure drug attributing to their enhanced solubility. The values are given in Table 2. DSC studies revealed that there is remarkable reduction in the enthalpy of microcrystals which could be due to the reduction in surface energy by adsorption of stabilizers over crystal surfaces. Reduction in enthalpy was 10 folds greater for ABZ-GK compared to ABZ-HG microcrystals inferring that gum karaya has high adherence on albendazole to form protective hydrophilic layer.

**Table 2:** DSC analysis (ABZ: Albendazole, GK:Gum karaya, HG:Hupu gum)

**Formulation**	**Melting point (°C)**	**Enthalpy change (- ∆H) (J/g)**
ABZ	218.76	3430
ABZ-GK	207.78	23.31
ABZ-HG	224.52	248.90

### XRD studies

**Figure 3:**
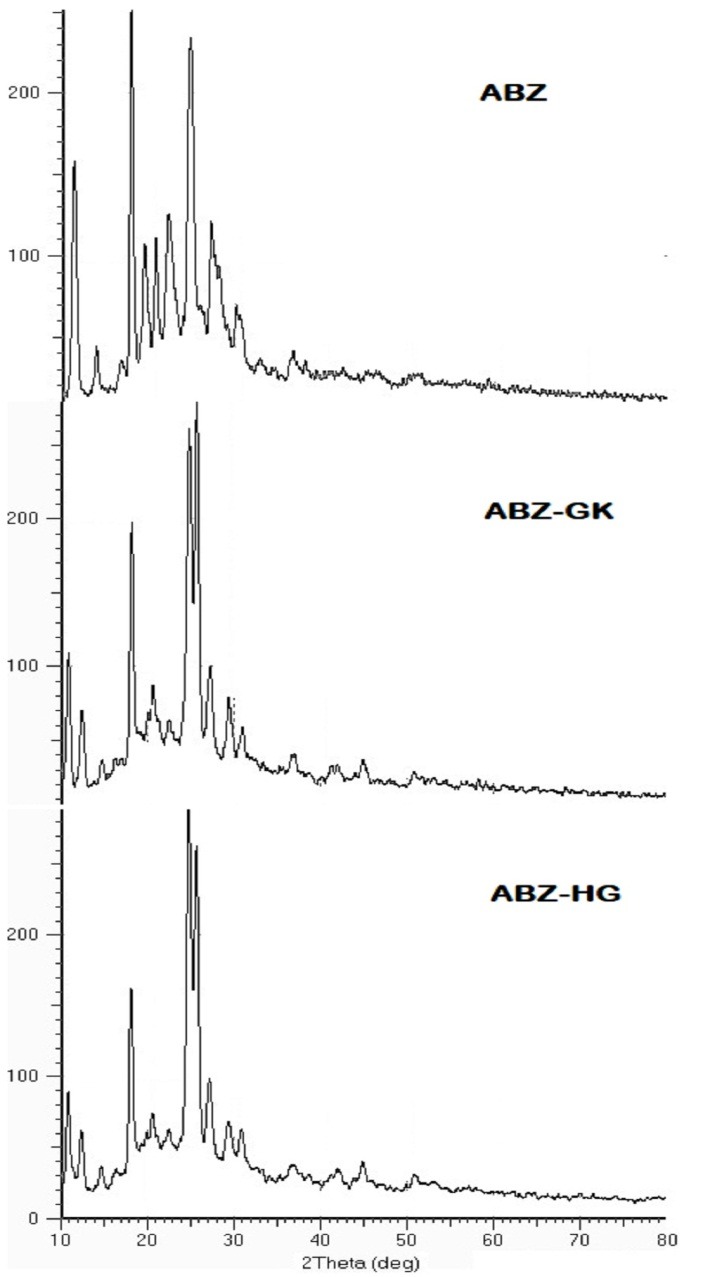
Comparative XRD of albendazole and microcrystals, (a) ABZ (b) ABZ-GK and (C) ABZ-HG (ABZ: Albendazole, GK:Gum karaya, HG:Hupu gum)

XRD studies were performed to investigate any changes in internal structure of the albendazole in the presence of stabilizing agents. The XRD patterns of pure drug, ABZ-GK and ABZ-HG were identical. Characteristic sharp peaks were observed at diffraction angles 10.4, 11.4, 19.5, 22.4, 23.24 and 24.83 as presented in Figure 3. XRD studies were performed to compare the crystalline nature of pure drug and the microcrystals. The intensity of peaks was reduced remarkably in the microcrystals. It was due to reduction in crystal size and extent of crystallinity of microcrystals. The results of XRD and DSC studies were combined to explain relation between crystallinity and enthalpy. It infers that the reduction in the enthalpy was due to change in crystalline behavior of albendazole in microcrystals.^23,24^

### Drug content

The practical yield of ABZ-GK and ABZ-HG microcrystals was observed to be 81.42 and 84.37% respectively. The percent drug content was found to be high for ABZ-GK (82.8%) than ABZ-HG (77.84%) due to higher surface interaction of albendazole with GK. No significant drug loss was observed during micronization. The formulations were evaluated for drug content to know any possible drug loss during preparation of microcrystals. The drug content was high for both the microcrystals with no significant drug loss. Drug present in ABZ-GK microcrystals was remarkably high compared to ABZ-HG microcrystals. The reason is the higher afffinity between albendazole and gum karaya.

### In-vitro dissolution studies

**Figure 4:**
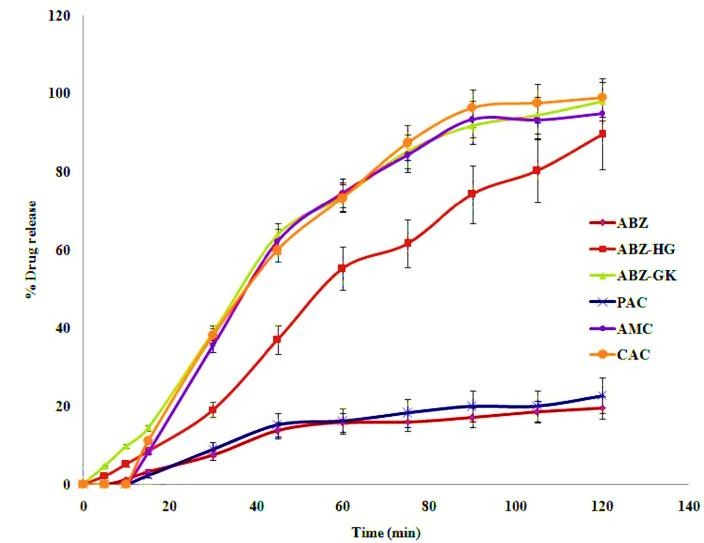
*In-vitro* dissolution profiles of albendazole microcrystals and capsules (values are represented as mean ± S.D., n=6) (ABZ: Albendazole, GK:Gum karaya, HG:Hupu gum, AMC: Albendazole microcrystal capsule, CAC: Commercial albendazole capsule, PAC: Pure albendazole capsule )

Drug release profiles from pure drug and the microcrystals are illustrated in Figure 4. *In-vitro* release studies were evident that albendazole microcrystals obtained from gum karaya had highest dissolution profiles followed by ABZ-HG microcrystals. It was also observed that the stabilizing agents have significant effect on the dissolution parameters (DE_60_, DE_90_, T_50_ and T_90_). The values are given in Table 3. The drug release rate of microcrystals was found to be three times faster compared to pure drug. The rate is highest for ABZ-GK microcrystals followed by ABZ-HG. Dissolution parameters also supported the results.

**Table 3:** Dissolution parameters of albendazole microcrystals and capsules (ABZ: Albendazole, GK:Gum karaya, HG:Hupu gum, AMC: Albendazole microcrystal capsule, CAC: Commercial albendazole capsule, PAC: Pure albendazole capsule )

**Dissolution parameter**		**ABZ**	**ABZ-GK**	**ABZ-HG**	**PAC**	**CAC**	**AMC**
**Dissolution efficiency (%)**	**DE**_60_	7.93 ± 1.05	38.45 ± 2.34	22.94 ± 2.01	11.82 ± 1.22	51.92 ± 1.42	53.01 ± 1.26
	**DE**_90_	9.54 ± 0.25	46.45 ± 1.29	30.04 ± 1.26	12.99 ± 0.88	57.82 ± 1.36	59.29 ± 2.23
**Time (min)**	**T**_50%_	-	36.93 ± 1.69	55.72 ± 2.37	-	38.11 ± 1.24	38.18 ± 1.26
	**T**_90%_	-	86.06 ± 2.98	-	-	84.49 ± 0.91	79.38 ± 2.94

Values are expressed as mean ± S.D. (n = 6)

*In-situ* micronization is very effective over other techniques with respect to increase in effective surface area without disturbing the internal structure of albendazole. Reduction in surface energy, increased hydrophilized effective surface area and enhanced wettability of albendazole were prime reasons duly extended by basic sugar components of gum karaya and hupu gum. GK and HG have similar sugar components but differ in their proportions. GK has more sugar moieties with more number of alkyl substituent that has higher affinity with hydrophobic surfaces.^25^ Hence surface adsorption, solubility and dissolution rate of ABZ-GK microcrystals were high compared to ABZ-HG microcrystals. Albendazole microcrystals containing gum karaya were considered as promised formulations.

### SEM analysis

ABZ-GK microcrystals that had shown highest solubility and dissolution efficiency were subjected to SEM analysis. In SEM analysis, the microcrystals were distributed uniformly with size of 41 µm. The SEM photographs had shown uniform size distribution and supported the results of microscopic particle size analysis.

### Evaluation of capsules

Albendazole microcrystals prepared using gum karaya were considered as optimized formulation and filled into size ‘1’capsules. The filled-in capsules (AMC) were evaluated alongside pure drug capsules and commercial capsules. The percentage weight variation was < 6.5 % indicating the uniformity in weight and the disintegration time was found to be less than 10 min (7.7 min and 7.9 min for PAC and AMC respectively) which are in compliance to the compendia limits.

### Drug release from capsules

The filled-in AMC capsules showed highest drug release rate followed by CAC and PAC as shown in Figure 4. The results were supported by the dissolution parameters as summarized in Table 3. PAC showed higher correlation in first-order drug release and obeyed Baker-Lonsdale kinetic model. The correlation coefficient values of AMC and CAC presented in Table 4 revealed that drug release patterns had good agreement in first-order release and Hixson-Crowell cube root kinetic model. Thus, albendazole microcrystals that had shown optimized properties were filled into capsules. The evaluation tests showed the weight variation and the disintegration time were in accordance with the compendia limits. AMC showed highest drug release. Dissolution efficiency was found to be more than 50% for AMC compared to PAC which showed less than 15% at 60 min. It is evident that PAC showed higher correlation for first-order release and obeying Baker-Lonsdale kinetic model. It can be inferred that the pure drug released into dissolution medium was based on its porosity. The correlation coefficient values for AMC and CAC were 0.9965 and 0.997 respectively and showed good agreement in first-order release and obeyed Hixson-Crowell cube root kinetic model indicating the mechanism of drug release was by erosion.^26^ The higher rate constant (K) values from Hixson-Crowell equation suggested faster dissolution rates for AMC and CMC compared to capsules containing albendazole alone.

**Table 4:** Release kinetics of albendazole from capsules (AMC: Albendazole microcrystal capsule, CAC: Commercial albendazole capsule, PAC: Pure albendazole capsule)

**Capsule code**	**Model**	**Correlation coefficient (r)**	**Rate constant (K)**
PAC	Baker-Lonsdale	0.9786	0.0001
CAC	Hixson-Crowell	0.9970	0.0289
AMC	Hixson-Crowell	0.9965	0.0206

## Conclusion

Microcrystals of albendazole with gum karaya and hupu gum were processed successfully *in-situ* micronization. Reduction of enthalpy, crystalline nature and increased solubility were duly contributed in the microcrystals with uniform size distribution, have shown highest drug release rate and exhibited extended stability. Moreover, the use of natural polysaccharides provides a better alternative for synthetic polymers due to their low toxicity and biocompatibility. Thus, *in-situ* micronization through solvent change method is able to avoid critical instability effects resulting from other micro-size reduction methods such as particle agglomeration and strong electrostatic attractions. Further this method provides a superior base for improving the solubility and dissolution of poorly soluble drug candidates on commercial scale.

## Acknowledgments

The authors are thankful to the management of Sree Vidyanikethan College of Pharmacy, Tiruapti, India; for providing the necessary facilities for the research work.

## Ethical Issues

Not applicable.

## Conflict of Interest

The authors declare no conflict of interests.
